# Evolutionary and Molecular Characterization of *liver-enriched gene 1*

**DOI:** 10.1038/s41598-020-61208-7

**Published:** 2020-03-06

**Authors:** Yanna Dang, Jin-Yang Wang, Chen Liu, Kun Zhang, Peng Jinrong, Jin He

**Affiliations:** Department of Animal Science, College of Animal Sciences, Zhejiang University, Hangzhou, PR China

**Keywords:** Phylogenetics, Agricultural genetics

## Abstract

*Liver-enriched gene 1* (*Leg1*) is a newly identified gene with little available functional information. To evolutionarily and molecularly characterize *Leg1* genes, a phylogenetic study was first conducted, which indicated that *Leg1* is a conserved gene that exists from bacteria to mammals. During the evolution of mammals, *Leg1s* underwent tandem duplications, which gave rise to *Leg1a*, *Leg1b*, and *Leg1c* clades. Analysis of the pig genome showed the presence of all three paralogs of pig *Leg1* genes (*pLeg1s*), whereas only *Leg1a* could be found in the human (*hLeg1a*) or mouse (*mLeg1a*) genomes. Purifying force acts on the evolution of *Leg1* genes, likely subjecting them to functional constraint. Molecularly, *pLeg1a* and its coded protein, pig LEG1a (pLEG1a), displayed high similarities to its human and mouse homologs in terms of gene organization, expression patterns, and structures. Hence, *pLeg1a*, *hLeg1a*, and *mLeg1a* might preserve similar functions. Additionally, expression analysis of the three *Leg1as* suggested that eutherian *Leg1as* might have different functions from those of zebrafish and platypus due to subfunctionalization. Therefore, *pLeg1a* might provide essential information about eutherian *Leg1a*. Moreover, a preliminary functional study using RNA-seq suggested that *pLeg1a* is involved in the lipid homeostasis. In conclusion, our study provides some basic information on the aspects of evolution and molecular function, which could be applied for further validation of *Leg1* using pig models.

## Introduction

*Leg1* (*liver-enriched gene 1*, or *C6orf58* homolog) is a newly identified gene with very little available functional information^1,2^. It is characterized by the presence of Domain of Unknown Function 781 (DUF781 or LEG1 domain) in its encoded protein^1,2^. *Leg1* was first identified in a zebrafish (*Danio rerio*) microarray study, in which it was named on the basis of its abundance in the liver^3^. Functional experiments later demonstrated that *Leg1* is involved in liver development, as knock-down of *Leg1* in embryos results in small liver phenotype due to blocked liver expansion^1^. Another functional study of the *Leg1* gene was performed in the platypus (*Ornithorhynchus anatinus*), in which the human *C6orf58* paralog *MLP* encodes monotreme lactation protein (MLP). MLP protein is a secreted protein that is enriched in milk, where it can exert antibacterial activity. Thus, it is presumed that MLP is related to the innate immunity of monotremes during the nipple-less delivery of milk to the hatchlings^2^.

Proteomic studies in eutherian species revealed that the *Leg1*-encoded N-glycosylated LEG1 protein is mainly present in saliva and seminal plasma^4,5^. However, no further functional studies were carried out in eutherian animals. Expression profiling analyses of mouse (*Mus musculus*) and human (*Homo sapiens*) *Leg1s* (*mLeg1* and *hLeg1*) reported in the Expression Atlas (www.ebi.ac.uk/gxa/home) showed that the gene is not expressed in the liver or mammary glands, in contrast to studies in monotremes and fishes, implying that eutherian *Leg1s* might have different biological functions. In addition, the preliminary evolutionary analysis revealed only one copy of *Leg1* in humans and mice, whereas the majority of other mammals harboured at least two *Leg1* gene copies. Two major clades, each of which consists of *C6orf58* orthologs or paralogs, could be identified for mammalian *Leg1*s in the phylogenetic tree, indicating possible gene duplication event in mammals^2^. Gene duplication resulted from whole genome duplication, unequal crossover, or segmental duplication is an important factor for speciation, adaptation, and gene family expansion^6,7^. The duplicated paralogs, which are then subjected to evolutionary selection, could conserve their original functions, acquire novel functions (neofunctionalization), maintain a specialized subfunction (subfunctionalization), or lose gene functions (pseudogenization)^8–10^. Since there might have been a duplication event during the evolution of the mammalian *Leg1* genes, orthologous *Leg1* genes must be cloned and characterized from closely related species to provide information about *hLeg1*, if there is the existence of functional constraint on the genes during evolution. Therefore, our study was conducted from two perspectives: 1) evolutionarily, the identification of orthologous genes in model organisms was carried out; 2) molecularly, it was determined whether the chosen orthologous genes show similar characteristics to *hLeg1*.

To accomplish these two goals, we initially conducted a comprehensive phylogenetic study using all available DUF781 domain harboring proteins (LEG1/LEG1L proteins hereafter), revealing that *Leg1* is a conserved gene that exists from bacteria to mammals. Moreover, during mammalian evolution, *Leg1* experienced tandem duplications that eventually gave rise to the *Leg1a*, *Leg1b*, and *Leg1c* paralogs. These *Leg1* genes are evolutionarily constrained, and in several species, *Leg1b* and *Leg1c* copies might have been pseudogenized, leaving *Leg1a* as the primary form of *Leg1* in eutherian genomes, especially in primates.

To study the functional role of *hLeg1a*, model organisms other than zebrafish (*Danio rerio*), *Caenorhabditis elegans*, and *Drosophila melanogaster* were needed, as no *Leg1* copy has been identified in the last two species. Hence, pigs (*Sus scrofa*) and mice might be better alternatives for elucidating the function of the *hLeg1a* gene. As the *mLeg1a* gene has been characterized^11^ as presenting only one functional copy, the construction of *mLeg1a* knockout mice is a straightforward and critical way to elucidate the function of *Leg1* in eutherians. The pig is not only an important livestock species but is also highly similar to humans in anatomy, physiology, and metabolism, making it an attractive alternative large animal model for human diseases^12^. Therefore, studying *pLeg1* could provide new insights into both agricultural and biomedical applications in addition to its biological mechanism. In contrast to *mLeg1a*, pigs have three *Leg1* gene copies (*pLeg1a*, *pLeg1b*, and *pLeg1c*). Though phylogenetic analysis shows *hLeg1a*, *mLeg1a*, and *pLeg1a* are orthologs, it remains to be determined which of these pig *Leg1* copies is molecularly relevant to *hLeg1a*. In this study, the cloning and characterization of the pig *Leg1* genes revealed that *pLeg1a* was the only one of the genes to be transcriptionally detectable. Additionally, *pLeg1a* has a similar expression pattern to *hLeg1a* and *mLeg1a*. Structural prediction also indicated that pLEG1a, hLEG1a, and mLEG1a are closely related. Finally, RNA-seq was performed to predict the potential function of the *Leg1* gene. The results showed that overexpression of *pLeg1a* affected certain biological processes (e.g., lipid homeostasis) and the level of *PPARγ*. Therefore, through our study, we provide some basic information regarding the evolution of the *Leg1* gene and demonstrate that *pLeg1a* is evolutionarily and molecularly close to *hLeg1a*, which could be applied for the further functional annotation of *hLeg1a* through the use of porcine models.

## Materials and Methods

### Construction of the phylogenetic tree

To retrieve the sequences for phylogenetic analysis, human (*Homo sapiens*) LEG1a (NP_001010905.1), mouse (*Mus musculus*) LEG1a (NP_080612.1), platypus (*Ornithorhynchus anatinus*) MLP (NP_001310705.1), and zebrafish (*Danio rerio*) LEG1s (NP_001093526.1, NP_998368.1) were used as queries to search against the non-redundant protein database using the phi-blast algorithm^13^ with iterated searches until no further significant hits were found. The obtained hits were initially screened based on an E-value < 0.005, and the redundant sequences, spliced variants, and hits with lengths that were too short were then removed. Then, the NCBI Genome, Ensembl, and UCSC Genome Browsers were used to search for additional annotated or predicted *Leg1* gene loci. If there was no *Leg1* information available in a species, the surrounding sequences according to synteny were subjected to GENSCAN^14^ for the prediction of potential protein-coding genes. Next, the obtained sequences were further screened for the presence of the DUF781 domain using CDD/SPARCLE^15^. Finally, 413 sequences with characteristic DUF781 domains (409 sequences have DUF781 as their sole identifiable domain; *Ochotona princeps* (XP_004587370.1) and *Meleagris gallopav*o (g5274.t1) LEG1s contain two DUF781 domains; *Bison bison bison* (GENSCAN_predicted_DUF781containing_peptide) and *Saccoglossus kowalevskii* (XP_006815645.1) LEG1s harbor predicted domains other than DUF781.) were included in subsequent studies (Supplementary spreadsheets 1 and 2). Based on the information provided by the Ensembl Gene Tree and NCBI annotations, vertebrate DUF781 domain containing proteins were named as LEG1 proteins, while those from invertebrates were designated as LEG1 like proteins (LEG1L). Correspondingly, the genes were named as *Leg1* and *Leg1l*.

To clearly illustrate *Leg1* and *Leg1l* evolution, the following representative species were chosen: primates (*Homo sapiens*, *Macaca mulatta*, *Microcebus murinus*), rodents (*Rattus norvegicus*, *Mus musculus*), Perissodactyla (*Equus caballus*), Artiodactyla (*Bos taurus*, *Ovis aries*, *Sus scrofa*), carnivores (*Canis lupus familiaris*, *Felis catus*), Lagomorpha (*Oryctolagus cuniculus*), Chiroptera (*Pteropus vampyrus*, *Myotis lucifugus*), *Echinops telfairi*, *Galeopterus variegatus*, *Loxodonta africana*, *Sarcophilus harrisii*, *Ornithorhynchus anatinus*, birds (*Gallus gallus*, *Taeniopygia guttata*, *Apteryx australis mantelli*), reptiles (*Alligator mississippiensis*, *Chrysemys picta bellii*, *Anolis carolinensis*, *Thamnophis sirtalis*), amphibian (*Xenopus tropicalis*, *Xenopus laevis*), lobe-finned fish (*Latimeria chalumnae*), 2 R ray-finned fish (*Lepisosteus oculatus*), 3 R ray-finned fish (*Takifugu rubripes*, *Danio rerio*), 4 R ray-finned fish (*Oncorhynchus mykiss*), cartilaginous fishes (*Callorhinchus milii*, *Rhincodon typus*), and Hemichordata (*Saccoglossus kowalevskii*). The coding sequences for *Leg1*/*pseudo-Leg1* and protein sequences were retrieved from the NCBI, Ensembl, or UCSC Genome Browser database (Supplementary spreadsheets 3 and 4).

Multiple sequence alignment was performed using Clustal Omega^16^ with default parameters. Maximum likelihood (ML) trees were then constructed using the MEGA7 toolbox^17^ with a bootstrap testing for 1,000 times. Protein trees were established using the JTT + G, while the DNA tree was generated via the T(92) + G method. The parameters were chosen based on the BIC and AIC values given by ModelTest-NG^18^ and MEGA7. Bayesian trees were established by using MrBayes 3.2.7a^19^.

Gene divergent time was estimated using the RelTime-ML^20^ in the MEGA toolbox according to the guideline^21^. The calibration times were retrieved from the TimeTree^22^.

The Gene Structure Display Server was employed to depict the organization of each *Leg1* gene organization by comparing the coding sequences against their respective genome sequences^23^.

### Microsynteny analysis

Microsyntenic analysis was adapted from a previous study^24^. Briefly, the protein-coding genes adjacent to *Leg1/Leg1l* were checked based on the available genome annotation data. The analysed species included eutherians, *Ornithorhynchus anatinus*, *Sarcophilus harrisii*, *Monodelphis domestica*, birds (*Gallus gallus*, *Taeniopygia guttata*, *Apteryx australis mantelli*), reptiles (*Alligator mississippiensis*, *Chrysemys picta bellii*, *Anolis carolinensis*, *Thamnophis sirtalis*), amphibians (*Xenopus tropicalis*, *Xenopus laevis*), fishes (*Danio rerio*, *Oncorhynchus mykiss*, *Oncorhynchus tshawytscha*, *Oncorhynchus kisutch*, *Salvelinus alpinus*, *Salmo salar*, *Labrus bergylta*, *Sinocyclocheilus anshuiensis*, *Sinocyclocheilus grahami*, *Sinocyclocheilus rhinocerous*, *Hippocampus comes*, *Takifugu rubripes*, *Oryzias latipes*, *Callorhinchus milii*, *Rhincodon typus*, *Latimeria chalumnae*), and invertebrates, diatoms, and bacteria. For those species with incomplete genome information, such as *Latimeria chalumnae*, in which the contigs -*leg1*-*soga3*-*echdc1* and *Ptprk*-*themis* are found in unmapped scaffolds, the synteny information will be partial and speculative based on closely related species. For 3 R and 4 R bony fish, *Leg1* duplication due to whole-genome duplication was considered to have occurred when two *Leg1* synteny groups were found in different chromosomes or linkage groups. To predict the absence of *Leg1* in some deuterostomes, the genes adjacent to *Leg1* according to the microsynteny of vertebrates and *Saccoglossus kowalevskii* were also subjected to BLAST searches against the genomes. When only *Leg1* was absent while other genes were found in the genomes, *Leg1* was considered to have been lost.

### Selection force dN/dS analysis

Multiple sequence alignment results generated by Clustal Omega were transferred to a codon alignment analysis using PAL2NAL^25^. Then, the Z-test of selection in MEGA7 software was used to test overall and pairwise selection force with an alternative hypothesis of dN/dS < 1, signifying purifying force. The paralogs of *Leg1* from each species were analysed using KaKs Calculator 2.0 to confirm the results from MEGA7 by using the GY-HKY, YN, and γ-YN methods^26^.

### RNA preparation and gene cloning

Tissues from the salivary glands (submandibular and parotid), heart, liver, spleen, lung, kidney, brain, small intestine, large intestine, and skeletal muscle of three female Rongchang pigs were collected and kindly provided by Dr. Lei Chen of the Chongqing Academy of Animal Science. These samples were stored in liquid nitrogen and subjected to RNA extraction using Total RNA Kit I (Omegabiotek) according to the manufacturer’s guideline. First-strand cDNA was then synthesized in a 25 μl volume using 1 μg RNA and d(T)18 primer according to the Promega M-MLV reverse transcriptase kit.

To obtain the full coding regions of the *pLeg1* genes, three pairs of primers were designed according to the predicted RNA sequences (*pleg1a*: XM_003121211.1, *pleg1b*: XM_021074892.1, *pleg1c*: XM_021084485.1) spanning the distance from start codons to the stop codons. 3′ rapid amplification of cDNA ends (RACE) was then performed to acquire the 3′ information for *pLeg1a*. Briefly, 1 μg RNA was reverse-transcribed using the 3′RACE oligo dT primer. Then, two rounds of nested PCR were carried out using the primers pairs 3RACEL1/pleg1a-3RACEGSP1, and 3RACEL2/pleg1a-3RACEGSP2. 5′RACE was performed using the SMARTer RACE 5′/3′ Kit (Clonetech). Two rounds of nested PCR were carried out via random priming of cDNA with the primer pairs pleg1a-5RACEGSP1/UPM(long) and pleg1a-5RACEGSP2/UPM(short). Two-step PCR reactions were all performed using Phusion High-Fidelity Polymerases (Thermo Fisher) with an annealing temperature of 60 °C. All the amplified fragments were gel-purified (Thermo Fisher) and sent to BGI Genomics for Sanger sequencing.

### Expression profiles of *pLeg1* genes

RNA samples were prepared from the tissues indicated above. Reverse-transcription PCR (RT-PCR) was first employed to detect the expression patterns of *pLeg1* genes. For each gene, two pairs of primers were designed. The PCR reactions (35 cycles of 94 °C for 30 s, 60 °C for 30 s, and 72 °C for 30 s or 1 min) were then performed in 25 μl volume with 2.5 U Taq (Takara), 1 μl cDNA, 400 nM each primer, 200 mM MgCl_2_, and 200 μM dNTPs. Subsequently, to obtain a more accurate expression results, quantitative real-time PCR (qRT-PCR) was performed in triplicate for each sample from all the three pigs using a similar protocol to a previous report^27^. The qRT-PCR results were analysed using the 2^−ΔΔCt^ method^28^.

### Plasmid construction

The eukaryotic expression plasmid for *pLeg1a* was constructed as follows. First, primers including *BamH*I and *Xho*I sites were used to amplify *pLeg1a* from salivary gland cDNA. Then, the amplified fragment was digested with *BamH*I and *Xho*I and cloned into *BamH*I and *Xho*I sites of pCAG-3 ×FLAG to construct pCAG-pLeg1a-3 ×FLAG.

### Structural prediction and clustering analysis

Protein structures were predicted using the Phyre 2 tool^29^ based on platypus MLP/LEG1c structure (PDB ID: 4V00). The ProCKSI server^30^ was then employed to compare the structures using the Vorolign algorithm^31^, and a clustering tree was generated.

### RNA-seq analysis

HEK293T cells were cultured in DMEM (HyClone) with 10% foetal bovine serum (HyClone) in a 6-well plate until reaching 80% confluency. Then, the cells were transfected with 3 μg of the pCAG-pLeg1a-3×FLAG vector or 3 μg of the empty pCAG-3 ×FLAG vector using Lipofectamine 3000 (Thermo Fisher). After 48 hours, the cells were harvested, and total RNA was prepared. Then, the RNA was sent to Novogene (Beijing) for library construction and sequencing. The libraries were constructed with mRNA and sequenced on the Illumina Hiseq X Ten platform. The obtained reads (Gene Expression Omnibus accession: GSE134920) were assigned directly to hg38 transcripts and analysed by using Salmon (https://combine-lab.github.io/salmon/)^32,33^. After quantification, differential gene expression was carried out using the DESeq2 package^34^ with the following parameters (*P*-value ≤ 0.05 and |log_2_ (fold change)| ≥ 1). GO and KEGG enrichment of differentially expressed genes was performed by using the Database for Annotation, Visualization and Integrated Discovery (DAVID)^35,36^.

### Ethical statement

All of the experimental procedures described in the paper followed the guidelines of China Council on Animal Care and were approved by the Animal Welfare Committee of Zhejiang University.

## Results

### Phylogenetic analysis of *Leg1* and *Leg1l*

First, the human, mouse, platypus, and zebrafish LEG1 protein sequences were used as queries for BLAST searches against the non-redundant protein database using the phi-BLAST algorithm. A total of 413 polypeptides with characteristic DUF781 domains were retained for further analysis. These polypeptides belonged to species from taxa including bacteria (Actinomecete and Proteobacteria), slime mold, diatom, invertebrates (Protozoa, Placozoa, Cnidaria, Echinodermata, Hemichordata), and vertebrates (except for Cyclostomata). Thus, these DUF781 domain encoding genes seem to be conserved across from prokaryotes to primates. However, in plants, fungi, many invertebrates (such as *Caenorhabditis elegans* and *Drosophila melanogaster*), Protochordata, and Cyclostomata, no positive hits for *Leg1/Leg1l* could be recovered using several gene prediction tools and alignment methods across various databases.

**Figure 1:**
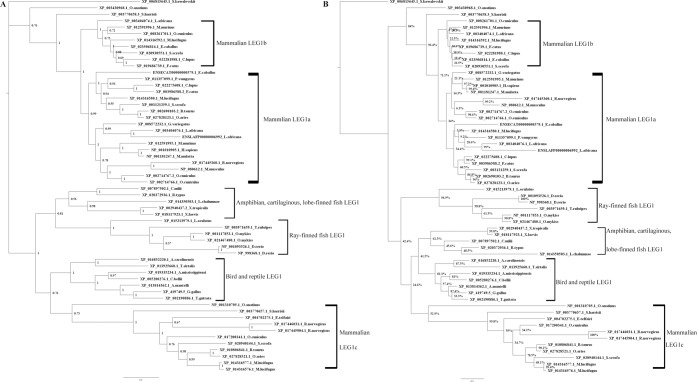
Phylogenetic analysis of LEG1 protein sequences in representative vertebrates performed using Bayesian (**A**) and ML methods (**B**). Node credibility is shown in A, and bootstrap values are shown in B. The two approaches produced a similar tree topology. Three mammalian LEG1 clades were generated. The one with human *C6orf58* homolog was named as LEG1a, and the clade with *M. murinus* paralog was labeled as LEG1b. The mammalian LEG1 clade grouped with other species is designated as LEG1c. The *Saccoglossus kowalevskii* LEG1L was used as an outgroup. The clustering results are labeled by the square brackets.

To explore the phylogenetic relationships of these LEG1/LEG1L proteins, all 413 identified proteins, or those from representative vertebrates, were subjected to phylogenetic analysis using the Maximal Likelihood and Bayesian approaches, which resulted in similar topologies (Fig. 1 and S1). According to the analysis, there are three distinct clades of LEG1 in mammals (Fig. 1 and S1). The clade with human and most other primate LEG1s was first designated the LEG1a clade. In primates, only *Microcebus murinus* exhibited two predicted functional *Leg1* genes. Thus, the other *Microcebus murinus* LEG1 was designated as LEG1b, and the clade including this sequence was designated as the LEG1b clade. Clustering analysis also demonstrated that the human pseudogenized *Leg1* gene could be grouped into the *Leg1b* clade using LEG1 coding sequences (Fig. S1D). The eutherian LEG1as and LEG1bs clustered together with prototherian and metatherian LEG1s to form a separated clade relative to the third LEG1 clade, which was designated as the mammalian LEG1c clade. The majority of eutherians presented a LEG1 copy in the LEG1a clade, except for *Daspypus novemcinctus* and *Echinops telfairi*, for which the only LEG1 copy was grouped in the LEG1b or LEG1c clades, respectively. Thus, the *Leg1a* gene might be the major functional gene in eutherians. Analysis of the mouse genome revealed that *mLeg1* is an orthologous gene of *hLeg1*^11^, and there is also a *pseudo-mLeg1a* (ENSMUST00000213962.1), as shown by the clustering analysis using *Leg1* coding sequences (Fig. S1D). Phylogenetic study classified the three pig LEG1 proteins into these three distinct clades, demonstrating that *pLeg1a* is an ortholog of both *hLeg1a* and *mLeg1a*. Only a few eutherians have maintained a copy that can be clustered with *Leg1c*. Interestingly, among the eutherian species, the *Rattus norvegicus* (Rodentia) and Bovidae species exhibit the *Leg1c* and *Leg1a* genes without *Leg1b* (Fig. S2). Among other species, the lobe-finned fish (*Latimeria chalunmae*) was grouped first with amphibians and then formed a separate clade with cartilaginous fishes, indicating the evolutionary linkage of amphibians and Crossopterygii^37^. In invertebrates, *Leg1l* was found in Hemichordata and Echinodermata, demonstrating a common evolutionary ancestor of these species with vertebrates as Deuterostomes.

**Figure 2:**
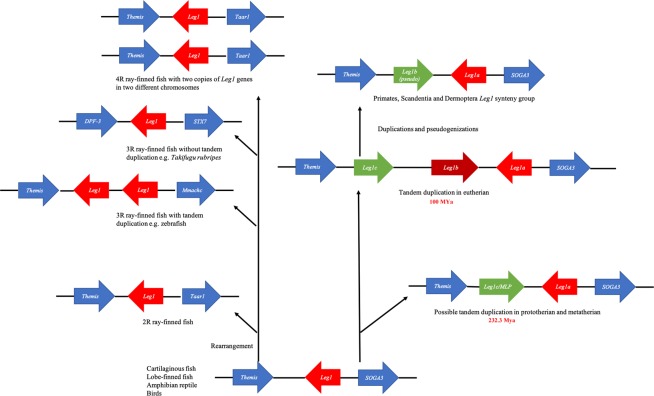
The genomic organization of *Leg1* and neighbouring genes during vertebrate evolution. The *Leg1* and neighbouring genes are represented with arrows whose direction indicates transcription orientation. The red arrows indicate the *Leg1*s in ray-finned fishes, cartilaginous fish, lobe-finned fish, amphibian, reptile, and bird; *Leg1a/Leg1b* in mammalian species. The blue arrows represent the neighbouring genes, while the green ones show either the pseudogenes or *Leg1c*s. The scheme is not depicted to scale, and not all of the genes in the region are shown.

Consistent with a previous report, most of the vertebrate *Leg1* genes resided within a microsynteny group between the *Themis* and *SOGA3* genes, except for those of 2 R ray-finned fish, 3R-/4R-teleosts, *Rattus norvegicus* and Bovidae^2^. To summarize the evolutionary history of the *Leg1* genes in vertebrates, molecular phylogeny in combination with synteny group analysis using the available LEG1 polypeptides was carried out. Figures 1 and 2 show that only one copy of *Leg1* remains in vertebrates after 1 R and 2 R whole-genome duplication events, since *Latimeria chalunmae* and *Lepisosteus oculatus* have only one copy of the *Leg1* gene. *Latimeria chalunmae* exhibited the same syntenic gene group found in amphibian, reptiles, birds, and mammals; however, chromosome rearrangement occurred in *Lepisosteus oculatus*, driving the *SOGA3* gene to a location approximately 10 Mb upstream of *Leg1* in the same linkage group. Additionally, among other bony fish species, only *Salmoninae*, *Sinocyclocheilus*, zebrafish (*Danio rerio*), *Hippocampus comes*, and *Labrus bergylta* harboured more than one copy of *Leg1*. Further microsyntenic analyses revealed that the *Leg1* copies of the first two taxa resulted from a whole-genome duplication event yielding two copies of *Leg1* genes on different chromosomes, while the other species experienced a tandem duplication event to yield an extra copy of *Leg1*.

### The *Leg1* gene is evolutionarily constrained

To determine whether *Leg1* genes are evolutionarily constrained, *Leg1* coding sequences (Supplementary spreadsheet 4) from representative vertebrates were analysed for the overall coverage of sequences using the Z-test of selection in the MEGA7 package. The outcome of the analysis showed a *P*-value near 0, indicating that there might be strong purifying selection on the whole vertebrate *Leg1* genes. Furthermore, to determine whether the *Leg1* gene may acquire a new function or preserve its current activity, within species *Leg1* paralogs were subjected to dN/dS calculation. Supplementary table S1 points out that nearly all of the duplicated pairs were subjected to a purifying force; however, there are still some exceptions, including *Rattus norvegicus Leg1c*, zebrafish *Leg1*, *Loxodonta africana Leg1a*, *Myotis lucifugus Leg1c*, and *Oryctolagus cuniculus Leg1c*. These results indicate that only a small proportion of recently duplicated *Leg1* sequences might have experienced neutral selection or positive selection (e.g., *Oryctolagus cuniculus* dN/dS > 1).

### Molecular cloning and characterization of *pLeg1*

**Figure 3:**
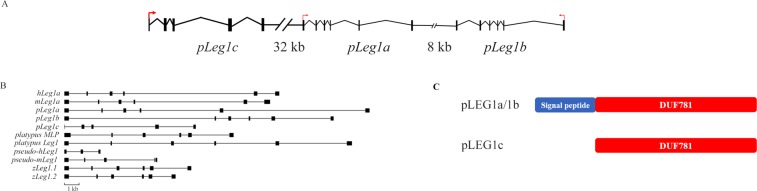
Analysis of the *pLeg1* genes and pLEG1 proteins. (**A**) Genetic structure and transcription orientation of pig *Leg1* genes. Red arrows indicate the possible transcription orientation. Black vertical boxes denote the exons of each *Leg1* gene. The distances between *pLeg1c* and *pLeg1a*, *pLeg1b* and *pLeg1a*, are also indicated. (**B**) Comparison of *Leg1* gene structures in human, mouse, pig, platypus, and zebrafish. Large variation of the gene structures could be noted in these *Leg1*s. The black vertical boxes are exons, while the horizontal lines show the introns. A scale bar is added below. (**C**) The predicted domains of pLEG1 proteins. Signal peptides (blue rectangle) are detected in pLEG1a and pLEG1b, while pLEG1c loses the domain. All three pLEG1s contain the characteristic DUF781/LEG1 domain (red rectangle) predicted by the CDD/SPARCLE.

Evolutionary analyses indicated that mammalian *Leg1a* orthologs have the highest probability of resembling the function of *hLeg1a*. As mouse *Leg1a* has been cloned and characterized previously, we identified the pig *Leg1a* gene in the current study^11^. Based on the information provided by the NCBI and Ensembl databases, three putative *pLeg1* genes were identified on chromosome 1 (LOC100511607, LOC100512146, LOC110259407), between the *THEMIS* and *SOGA3* gene loci. Herein, we designated these genes *pLeg1a*, *pLeg1b*, and *pLeg1c* according to their phylogenetic grouping. The lengths of the predicted coding regions of these three genes were 1,014 bp, 1,020 bp, and 684 bp, respectively, spanning a region of ~80 kb (Fig. 3A). After sequencing the genes using salivary gland RNA, the open reading frame of *pLeg1a* (GenBank Accession no. MN481509) was found to contain 1,014 bp with a sequence identical to the XM_003121211.1. Information for the other two *pLeg1s* could not be obtained by molecular cloning. Thus, in the subsequent analysis, the predicted mRNA and protein sequences were used (*pLeg1b*: XM_021074892.1/XP_020930551.1, *pLeg1c*: XM_021084485.1/XP_020940144.1). Similar to their human, mouse, and zebrafish counterparts, *pLeg1a* and *pLeg1b* have six exons, while *pLeg1c* only has 5 exons (Fig. 3B).

The *pLeg1* genes encode three polypeptides with lengths of 339 aa, 337 aa, and 227 aa. These polypeptides all have a characteristic Domain of Unknown Function 781 (DUF781/LEG1, pfam05612, accession no. cl05272) domain (Fig. 3C). Sequence comparison showed that pLEG1a and pLEG1b are closest to each other with a 66%/79% identity/similarity, while pLEG1c was more distant from the other two pLEG1 proteins, with 19%/32% and 18%/31% identity/similarity. Pairwise comparison also indicated that pLEG1a showed the highest identity/similarity to human LEG1a (Table 1).

**Table 1:** Pairwise comparison of LEG1 proteins from the indicated species.

	hLEG1a	mLEG1a	pLEG1a	pLEG1b	pLEG1c	zLEG1.1	zLEG1.2	platypus LEG1	platypus LEG1c/MLP
hLEG1a									
mLEG1a	54/72^a^								
pLEG1a	61/75	55/69							
pLEG1b	61/72	54/68	66/79						
pLEG1c	15/29	18/31	19/32	18/31					
zLEG1.1	32/50	31/48	29/48	30/48	15/27				
zLEG1.2	31/51	31/48	28/48	28/49	14/26	90/93			
platypus LEG1	44/58	42/58	46/60	45/60	17/28	33/50	32/48		
platypus LEG1c/MLP	29/45	31/45	29/47	29/47	16/28	28/47	27/46	31/49	

^a^Data are presented as identity/similarity × 100.

### Expression profile of *pLeg1* genes

**Figure 4:**
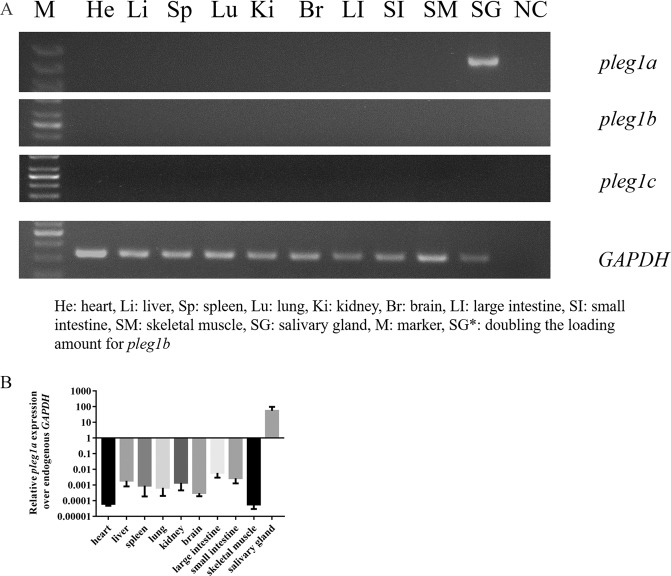
Expression patterns of *pLeg1* genes in various tissues. (**A**) RT-PCR analysis of *pLeg1a*, *pLeg1b*, and *pLeg1c* in pig tissues showed that *pLeg1a* was specifically expressed in the salivary gland (upper panel). *pLeg1b* and *pLeg1c* signals could not be obtained in these tissues (the middle two panels). *GAPDH* was used as internal control (lower panel). (M, marker. He, heart. Li, liver. Sp, spleen. Lu, lung. Ki, kidney. Br, brain. LI, large intestine. SI, small intestine. SM, skeletal muscle. SG, salivary gland. NC, negative control). (**B**) The expression pattern of *pLeg1a* was confirmed by qRT-PCR analysis using the expression level of *GAPDH* as reference. Data are presented as mean ± SEM.

To determine the expression pattern of these three *pLeg1* copies, RT-PCR was first employed, demonstrating that only *pLeg1a* was specifically detectable in the salivary glands, while there was no signal for *pLeg1b* or *pLeg1c* (Fig. 4A and S3). Then, qRT-PCR was performed to confirm the RT-PCR results. As shown in Fig. 4B, *pLeg1a* was highly expressed in the salivary glands, and *pLeg1b/1c* were undetectable in various tissues. BLAST was also employed using the *pLeg1* sequences as queries against the EST database, and only hits for *pLeg1a* were found, mainly from the salivary glands.

### Structural prediction implies that LEG1 might retain a conserved function

**Figure 5:**
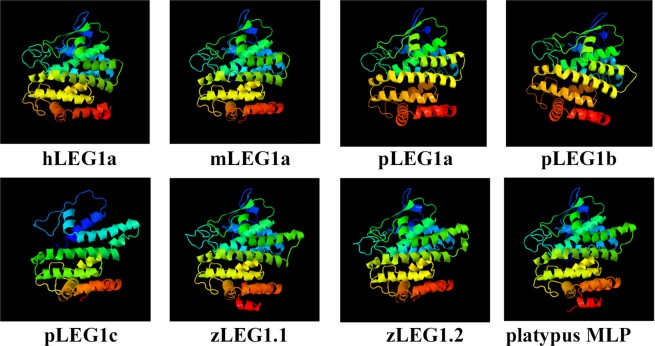
Structural comparison of LEG1 proteins from different species. The platypus MLP protein structure was retrieved from the PDB (4V00), while the others were predicted using Phyre 2. The colors are in rainbow order with red and blue colors indicate the N- and C- termini of LEG1, respectively. All LEG1 proteins exhibit the similar structural prediction result expect for pLEG1c, which is slightly different from others due to the lack of the signal peptide.

Currently, only the platypus LEG1c/MLP protein has been structurally resolved^38^. Based on the information provided, other LEG1 protein structures from representative vertebrates were predicted using the Phyre2 tool. The resulting prediction showed similar structures of all LEG1 proteins except for pLEG1c (Fig. 5). To quantify the similarities between different LEG1 proteins, the predicted structural information was submitted to the ProCKSI server, and clustering was finally established. The majority of these LEG1 proteins were clustered in accordance with the phylogenetic tree using sequence information (Fig. S4). The structural tree presented two major branches, in one of which teleost LEG1s were first grouped with platypus MLP and then clustered together with bird, amphibian, and reptile LEG1s. Eutherian LEG1c, together with rat LEG1a and dog LEG1b, was also grouped within this branch. The other branch mainly contained eutherian LEG1 proteins (LEG1a and LEG1b), although LEG1 from the metatherian *Sarcophilus harrisii* was in this branch as well. These results suggested that the structures of LEG1 proteins are highly similar to each other and were analogous to the phylogenetic results. Additionally, hLEG1a, mLEG1a, and pLEG1a, and pLEG1b are structurally closely related, indicating they may possess similar functions. Therefore, mouse and pig are good models for studying the function of *hLeg1* gene.

### Preliminary functional prediction of *pLeg1a* using RNA-seq

**Figure 6:**
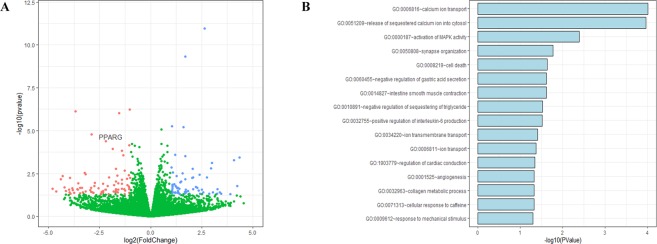
RNA-seq analysis of cells with overexpressing *pLeg1a*. (**A**) Volcanic plot of genes identified through RNA-seq. Each dot represents an individual gene. Red dots denote downregulated DEGs, while blue dots represent upregulated DEGs. *PPARγ* is also shown in the plot. (**B**) Enrichment analysis indicates that several biological processes are significantly affected by the DEGs.

To functionally predict the role of *pLeg1a*, HEK293T cells were transfected with pCAG-pLeg1a-3 ×FLAG and the empty vector for RNA-seq (GSE134920). A total of 152 genes were differentially expressed (|log_2_Fold Change| ≥ 1 and *P*-value ≤ 0.05), among which 85 DEGs were downregulated, and 67 DEGs were upregulated. One of these DEGs was *PPARγ*, which plays a role in the regulation of lipid metabolism and adipocyte differentiation (Fig. 6A). Enrichment analysis showed that some DEGs were enriched in the negative regulation of triglyceride sequestering. In addition, some DEGs were enriched in calcium associated biological processes (Fig. 6B). These results indicate that mammalian *Leg1* genes might be involved in lipid and calcium homeostasis.

## Discussion

In the current study, we described the evolution of *Leg1*/*Leg1l* and molecularly characterized *pLeg1* genes, including a phylogenetic study and analysis of their cDNA sequences, expression profiles, exon-intron organization, predicted structures, and potential associated molecular processes. We propose that as genes with unknown function in eutherian species, *pLeg1a*, *mLeg1a*, and *hLeg1a* might share similar functions indicating that pigs and mice are good models for studying *hLeg1a*.

An initial BLAST analysis showed that *Leg1/Leg1l* exists from prokaryotes to mammals, demonstrating that the *Leg1/Leg1l* gene is conserved. However, in prokaryotes, plants, and Protostomia, copies of *Leg1/Leg1l* were detected in only a few species. One possible reason for this result might have been that *Leg1/Leg1l* could not be found in these species due to poor genome annotations. For example, in the Cyclostomata genome, it was not only *Leg1* but also genes such as *Soga3*, *Themis*, and *Echdc1* could not be found. However, there was also no signal observed in some well-characterized organisms, such as *Drosophila melanogaster*, *Caenorhabditis elegans*, *Arabidopsis thaliana*, indicating that *Leg1/Leg1l* has been lost and might not be essential in these species. Within Deuterostomia, *Leg1/Leg1l* was identified in the Echinodermata, Hemichordata, and Gnathostomata, but not in Urochordata, Cephalochordata, Cyclostomata, or Xenoturbella^39^. Molecular phylogeny indicates that hemichordates and echinoderms are closely related and form a supraphylum referred to as Ambulacraria^40^, in which the *Leg1/Leg1l* gene could be found in this clade (Fig. S1). In Protochordata, we used the adjacent genes according to the information from vertebrates and hemichordates as queries to search the genomes of its members. The presence of these syntenic genes in combination with the absence of a *Leg1/Leg1l* hit strongly suggested that *Leg1/Leg1l* has been lost in Protochordata. Therefore, during chordate evolution, *Leg1* might have been lost in protochordates and maintained in jawed vertebrates. This result suggests that *Leg1* may be relevant to specific biological mechanisms or behaviours in jawed vertebrates.

Analysis of the synteny of *Leg1* in jawed vertebrates revealed that cartilaginous fish, lobe-finned fish, and tetrapods exhibit a conserved organization in which *Leg1* resides between the *Themis* and *SOGA3* genes, while in Actinopterygii, genomic rearrangements have occurred (Fig. 2). Actinopterygii and Sarcopterygii diverged approximately 440 million years ago (Mya)^41^, and *Lepisosteus oculatus* is regarded as a bridge connecting teleost and tetrapod species. The syntenic organization has changed in *Lepisosteus oculatus*, suggesting a possible chromosomal rearrangement in the common ancestor of Actinopterygii. This rearrangement was not due to the 2 R genome duplication because evidence suggests that the 2 R genome duplication took place before the divergence of jawed and jawless vertebrates^42,43^. It is notable that most 3 R ray-finned fish only have one copy of *Leg1*, despite the teleost-specific whole-genome duplication^44–46^. Thus, the *Leg1* gene underwent nonfunctionalization after 3 R, which is a common outcome of duplication events^47^. There were also some exceptions in 3 R ray-finned fish such as *Labrus bergylta*, zebrafish (*Danio rerio*), and *Hippocampus comes*, in which two tandemly linked *Leg1* copies could be found (Fig. S1). Local tandem duplication may be a better explanation for this phenomenon. Detailed analysis of Salmoninae (*Oncorhynchus mykiss*, *Oncorhynchus tshawytscha*, *Oncorhynchus kisatch*, *Salvelimus alpinus*, *Salmo salar*), *Sinocyclocheilus anshuensis*, and *Sinocyclocheilus grahami* revealed the presence of multiple copies of *Leg1* located on different chromosomes. This phenomenon is most likely due to 4 R genome duplication in Salmonids and Cyprinids^48^.

Most Sarcopterygii and tetrapod species (birds, reptiles, and amphibians) exhibit preservation of the original syntenic organization of *Leg1* with only one *Leg1* copy; however, in mammals, multiple copies of *Leg1* emerged again (Fig. 2), resulting in the formation of three *Leg1* clades. In one clade, platypus *MLP* groups with a few other mammalian *Leg1s* in addition to bird and reptile *Leg1s*, suggesting an early duplication event before the divergence of eutherians from other mammals. Then, another duplication drove the formation of *Leg1a* and *Leg1b*. The estimated duplication time for *Leg1c* from other *Leg1*s was about 232.2 Mya, which is earlier than the divergence of eutherians from proto- and metatherians. Then, another duplication splitting *Leg1a* and *Leg1b* occurred 100 Mya, which is around the divergence of eutherian species (Fig. 2)^22^. As a consequence, proto- and metatherian *Leg1s* are clustered as an outgroup to eutherian *Leg1a* and *Leg1b*. As shown in Fig. S1, *Leg1a* is present in nearly all mammalian species, while some species or the majority of mammalian species lack *Leg1b* or *Leg1c*, respectively. In platypus, MLP has been suggested to exhibit antibacterial activity in the nipple-less delivery of milk to hatchlings^2^. However, this mechanism is not needed by eutherian species. Therefore, we speculate that *Leg1b* and especially *Leg1c* are not as essential as *Leg1a* and that they experienced nonfunctionalization and were lost during evolution in most eutherian species. A previous study established a phylogenetic tree involving genome information for 49 vertebrates^2^, in which platypus, *Sarcophilus harrisii*, *Cavia Porcellus*, *Bos taurus*, and *Ovis aries* were grouped together. However, the authors suggested that the grouping did not reflect the evolution of these species. In our study, after adding information from other mammals, we concluded that platypus *MLP* is a paralog of platypus *C6orf58*, and that *MLP/Leg1c* may present different functions in mammals.

Primate, Scandentia, and Dermoptera form a clade known as Euarchonta, which presents a close relationship to Glires^49–52^. The analysis of primates, *Tupaia chinensis*, and *Galeopterus variegatus* genomes revealed no *Leg1c*, and only *Microcebus murinus* presented a copy of *Leg1b*, which was significantly different from those found in Glires. Therefore, during the evolution of Euarchonta, *Leg1b* and *Leg1c* were lost. The debris of these nonfunctionalization events can still be observed in the human and *Galeopterus variegtus* genomes, as pseudogenes are clustered in the *Leg1b* clade (Fig. S1D). The Glires genomes are quite diverse; e.g., there are multiple copies of *Leg1* in the *Oryctolagus cuniculus* genome and several rodent genomes, while only *Leg1a* is detected in genus *Mus*. A possible reason for this situation might be that multiple genome alterations have taken place in this clade, resulting in extreme species diversity, especially in rodents^53^. Analysis of Laurasiatheria showed that in Carnivora and Cetacea, only *Leg1a* and *Leg1b* are present in the genome, while *Leg1c* has been lost. Suidae and Perissodactyla exhibit all 3 copies of *Leg1*, indicating the preservation of the original syntenic organization after *Leg1* duplication. Among these species, Bovidae species only present *Leg1a* and *Leg1c* copies. Detailed genome analysis indicated that a possible chromosomal inversion with one break site between *Leg1c* and *Leg1a* drove the loss of *Leg1b*, with another break site residing between the *SNAP91* and *Ripply 2* genes (Fig. S2). Thus, before inversion, the order of the genes should have been *SNAP91*-*Ripply 2* -*CyB5R4*-(…)-*SOGA3*-*Leg1a*-*Leg1b*-*Leg1c*-*Themis*, which then became *SNAP91*-*Leg1a*-*SOGA3*-(…)-*CYB5R4*-*Ripply 2*-*Leg1c*-*Themis* in Bovidae. A similar phenomenon can be found in the *Rattus norvegicus* genome (Fig. S2); however, due to complex genome alterations, the precise mechanism resulting in *Rattus norvegicus* synteny needs to be further studied.

To test how evolutionary forces act on the *Leg1* genes, we performed an overall Z-test of selection on *Leg1* sequences from representative vertebrates. Strong purifying selection was suggested by the test, implying that the *Leg1* genes have probably maintained their function during evolution. As paralogs may exhibit different fates after duplication (neofunctionalization, subfunctionalization, and pseudofunctionalization/nonfunctionalization^54^), we evaluated the dN/dS ratios between paralogous *Leg1* genes within each species. The results shown in Table S1 indicate that most of the duplicates are functionally constrained (dN/dS < 1), with a few exceptions (e.g., rabbit (dN/dS > 1) and zebrafish (dN/dS = 1), indicating positive and neutral selection, respectively). Previous *Leg1* functional studies were only carried out in platypus and zebrafish^1,2,55,56^. In these studies, different patterns of expression were observed, indicating that subfunctionalization might take place in these paralogs, with each *Leg1* copy preserving some aspects of its parental gene functions^9,57,58^. In addition, *hLeg1a*, *mLeg1a*, and *pLeg1a* show significantly distinct expression patterns from their platypus and zebrafish homologs, strongly suggesting subfunctionalization. In summary, our evolutionary analysis indicates that *mLeg1a*, *pLe*g1a, and *hLeg1a* are evolutionarily closely related and may retain the same functions.

Next, we cloned and characterized pig *Leg1* genes to demonstrate the molecular similarities between *hLeg1a* and *pLeg1a*. Our experiment showed that *pLeg1a* is highly similar to human and mouse homologs in terms of expression, and structure. Three *Leg1* copies were identified on pig chromosome 1 between *Themis* and *SOGA3*, spanning a region of ~80 kb. *pLeg1a* and *pLeg1b* have six exons, similar to their human and mouse counterparts. However, *pLeg1c* only has 5 exons (Fig. 3). Among these currently identified LEG1 proteins, pLEG1a shows higher similarity/identity with hLEG1a than does mLEG1a, despite a greater evolutionary distance (Table 1 and Fig. 1). As shown by previous predictions, the characteristic DUF781 domain follows the signal peptide^1,2,11^, which could be detected in all pig LEG1 proteins except for pLEG1c. Additionally, pLEG1c only shows 19% and 18% sequence identity to pLEG1a and pLEG1b, respectively. These results suggest that *pLeg1c* is evolutionarily divergent from its paralogs. Transcriptional analysis showed that *pLeg1a* is specifically expressed in salivary glands, whereas no signal was detected in these tissues for *pLeg1b* or *pLeg1c*. Our results are consistent with those of a microarray analysis demonstrating that *pLeg1a* is highly expressed in the submandibular gland^59^. Hence, pig and mouse studies have produced contrary results to those obtained in zebrafish and platypus, in which *Leg1* genes are expressed in the liver. Thus, it is unlikely that mammalian *Leg1a* plays a role in liver development. Interestingly, *Leg1c/MLP* could be detected in the platypus salivary gland. Therefore, expression analysis suggests that subfunctionalization of *Leg1* genes has occurred between mammals and fish. Finally, the structural prediction and clustering analysis using structural information were conducted. The results showed that hLEG1a, mLEG1a, and pLEG1a proteins are highly similar to each other structurally, implying a close functional relationship (Fig. 5 and Fig. S4). Therefore, the above experiments provided some basic evidence that *pLeg1a*, *mLeg1a*, and *hLeg1a* are functionally related.

There are generally two ways of studying gene function: loss of function and gain of function analyses. In this study, we performed an overexpression experiment by transient transfection of HEK293T cells using the *pLeg1a* expression plasmid to determine which proteins or biological processes would be affected. RNA-seq combined with enrichment analysis showed that several calcium and lipid-related pathways were involved. Among these pathways, we observed that *PPARγ* displayed significant downregulation (Fig. 6). As previous studies indicate that *PPARγ* plays a vital role in lipid homeostasis^60^, it is likely that the *Leg1a* gene also participates in lipid metabolism in mammals.

In conclusion, we cloned and characterized *pLeg1a* for the first time and demonstrated that it shows high similarity to *hLeg1a* and *mLeg1a* from evolutionary and molecular perspectives. Additionally, *pLeg1a* overexpression would result in the alteration of *PPARγ* and lipid homeostasis according to functional prediction using RNA-seq. Thus, *pLeg1a* might be an excellent model for investigating the function of *Leg1* genes in mammals in future studies.

## Supplementary information

Supplementary file.

Supplementary file 2.

Supplementary spreadsheet 1.

Supplementary spreadsheet 2.

Supplementary spreadsheet 3.

Supplementary spreadsheet 4.

## Data Availability

The materials used in the work are available upon contacting the authors.
