# Supplementary material for: Evolutionary and Molecular Characterization of liver-enriched gene 1
Source: Sci Rep. 2020 Mar 6;10:4262. doi: 10.1038/s41598-020-61208-7 (PMC7060313; doi:10.1038/s41598-020-61208-7)
Supplement: Supplementary file 1 — Supplementary file. [file 41598_2020_61208_MOESM1_ESM.pdf]

**Title: Evolutionary and Molecular Characterization of *liver-enriched gene 1***

**Authors:** Yanna Dang[1], Jin-Yang Wang[1], Chen Liu[1], Kun Zhang[1], Peng Jinrong[1], Jin He[1*]

**Affiliations**

[1] Department of Animal Science, College of Animal Sciences, Zhejiang University, Hangzhou, PR China

[*]Corresponding author: hejin@zju.edu.cn

## Additional information

**Fig. S1** Phylogenetic analysis of LEG1/LEG1L protein sequences. A. Maximum likelihood with JTT + G method was used to construct the unrooted phylogenetic tree. Bootstrap values greater than 60% are shown in the internal nodes. Different taxonomic units are highlighted with different colours. Protein entries from representative vertebrates are highlighted in red for their accession no. and species names. B and C. Phylogenetic trees with collapsed branches established using ML and Bayesian methods, respectively, were shown. D. Clustering analysis of *Leg1* coding sequences showing the grouping of some pseudogenes that are highlighted in red.

A

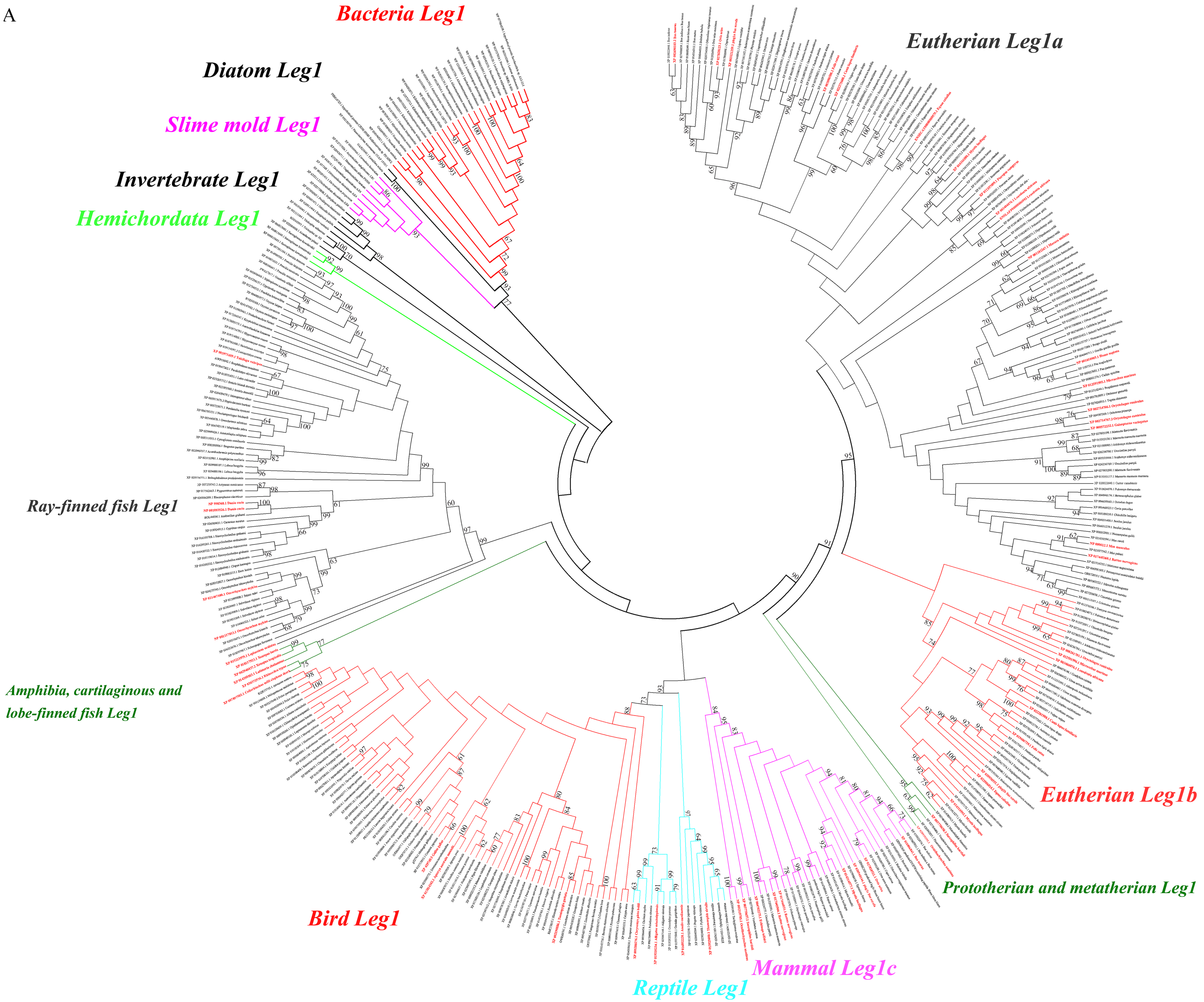

Bacteria Leg1
Diatom Leg1
Slime mold Leg1
Invertebrate Leg1
Hemichordata Leg1
Ray-finned fish Leg1
Amphibia, cartilaginous and
lobe-finned fish Leg1
Bird Leg1
Reptile Leg1
Mammal Leg1c
Prototherian and metatherian Leg1
Eutherian Leg1b
Eutherian Leg1a

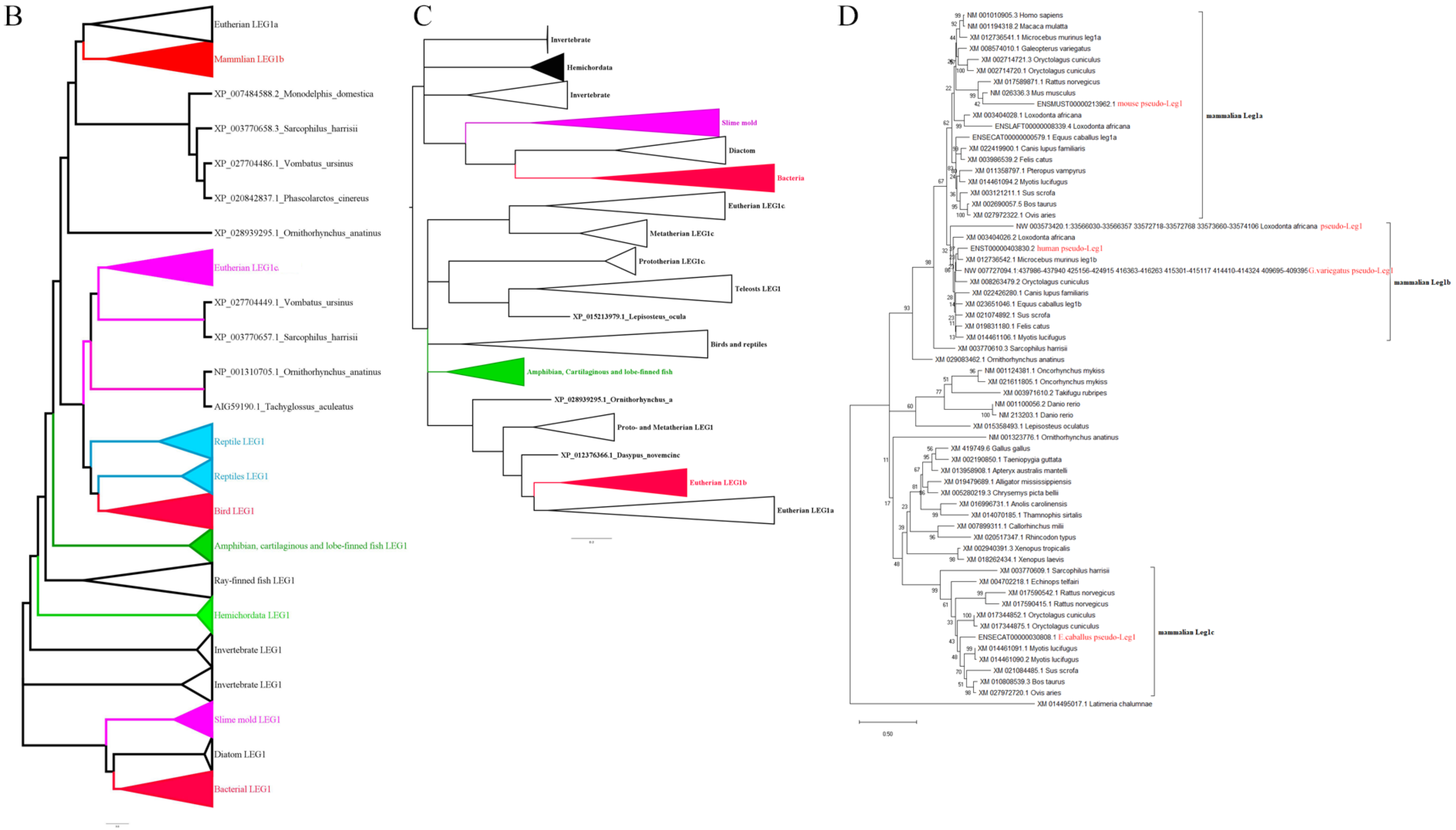
B
Eutherian LEG1a
Mammlian LEG1b
XP_007484588.2_Monodelphis_domestica
XP_003770658.3_Sarcophilus_harrisii
XP_027704486.1_Vombatus_ursinus
XP_020842837.1_Phascolarctos_cinereus
XP_028939295.1_Ornithorhynchus_anatinus
Eutherian LEG1c
XP_027704449.1_Vombatus_ursinus
XP_003770657.1_Sarcophilus_harrisii
NP_001310705.1_Ornithorhynchus_anatinus
AIG59190.1_Tachyglossus_aculeatus
Reptile LEG1
Reptiles LEG1
Bird LEG1
Amphibian, cartilaginous and lobe-finned fish LEG1
Ray-finned fish LEG1
Hemichordata LEG1
Invertebrate LEG1
Invertebrate LEG1
Slime mold LEG1
Diatom LEG1
Bacterial LEG1
C
Invertebrate
Hemichordata
Invertebrate
Slime mold
Diactom
Bacteria
Eutherian LEG1c
Metatherian LEG1c
Prototherian LEG1c
Teleosts LEG1
XP_015213979.1_Lepisosteus_ocula
Birds and reptiles
Amphibian, Cartilaginous and lobe-finned fish
XP_028939295.1_Ornithorhynchus_a
Proto- and Metatherian LEG1
XP_012376366.1_Dasypus_novemcinc
Eutherian LEG1b
Eutherian LEG1a
D
NM 001010905.3 Homo sapiens
NM 001194318.2 Macaca mulatta
XM 012736541.1 Microcebus murinus leg1a
XM 008574010.1 Galeopterus variegatus
XM 002714721.3 Oryctolagus cuniculus
XM 002714720.1 Oryctolagus cuniculus
XM 017589871.1 Rattus norvegicus
NM 026336.3 Mus musculus
ENSMUST00000213962.1 mouse pseudo-Leg1
XM 003404028.1 Loxodonta africana
ENSLAFT00000008339.4 Loxodonta africana
ENSECAT00000000579.1 Equus caballus leg1a
XM 022419900.1 Canis lupus familiaris
XM 003986539.2 Felis catus
XM 011358797.1 Pteropus vampyrus
XM 014461094.2 Myotis lucifugus
XM 003121211.1 Sus scrofa
XM 002690057.5 Bos taurus
XM 027972322.1 Ovis aries
mammalian Leg1a
NW 003573420.1:33566030-33566357 33572718-33572768 33573660-33574106 Loxodonta africana pseudo-Leg1
XM 003404026.2 Loxodonta africana
ENST00000403830.2 human pseudo-Leg1
XM 012736542.1 Microcebus murinus leg1b
NW 007727094.1:437986-437940 425156-424915 416363-416263 415301-415117 414410-414324 409695-409395G.variegatus pseudo-Leg1
XM 008263479.2 Oryctolagus cuniculus
XM 022426280.1 Canis lupus familiaris
XM 023651046.1 Equus caballus leg1b
XM 021074892.1 Sus scrofa
XM 019831180.1 Felis catus
XM 014461106.1 Myotis lucifugus
mammalian Leg1b
XM 003770610.3 Sarcophilus harrisii
XM 029083462.1 Ornithorhynchus anatinus
NM 001124381.1 Oncorhynchus mykiss
XM 021611805.1 Oncorhynchus mykiss
XM 003971610.2 Takifugu rubripes
NM 001100056.2 Danio rerio
NM 213203.1 Danio rerio
XM 015358493.1 Lepisosteus oculatus
NM 001323776.1 Ornithorhynchus anatinus
XM 419749.6 Gallus gallus
XM 002190850.1 Taeniopygia guttata
XM 013958908.1 Apteryx australis mantelli
XM 019479689.1 Alligator mississippiensis
XM 005280219.3 Chrysemys picta bellii
XM 016996731.1 Anolis carolinensis
XM 014070185.1 Thamnophis sirtalis
XM 007899311.1 Callorhinchus milii
XM 020517347.1 Rhincodon typus
XM 002940391.3 Xenopus tropicalis
XM 018262434.1 Xenopus laevis
XM 003770609.1 Sarcophilus harrisii
XM 004702218.1 Echinops telfairi
XM 017590542.1 Rattus norvegicus
XM 017590415.1 Rattus norvegicus
XM 017344852.1 Oryctolagus cuniculus
XM 017344875.1 Oryctolagus cuniculus
ENSECAT00000030808.1 E.caballus pseudo-Leg1
XM 014461091.1 Myotis lucifugus
XM 014461090.2 Myotis lucifugus
XM 021084485.1 Sus scrofa
XM 010808539.3 Bos taurus
XM 027972720.1 Ovis aries
mammalian Leg1c
XM 014495017.1 Latimeria chalumnae
0.50

**Fig. S2** Gene organization of *Leg1* and neighbouring genes in Bovidae and *Rattus norvegicus* genome. The arrow directions indicate the predicted transcription orientations.

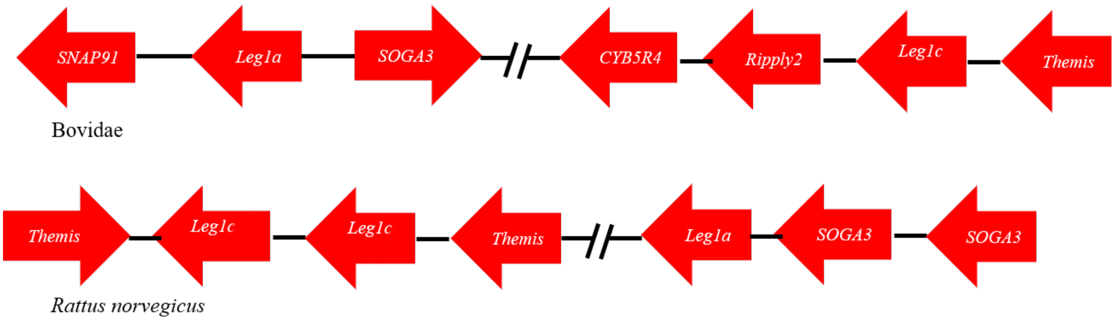

**Fig. S3** PCR testing of the efficiency of primers used in the expression profile. For each gene two pairs of primers were used (1 and 2, respectively). The substrates are nc ($H_2O$), WT (WT pig genome), and P (plasmid: pCAG-pleg1a-3 × FLAG, pCAG-pleg1b-3 × HA, pCAG-pleg1c-3×FLAG).

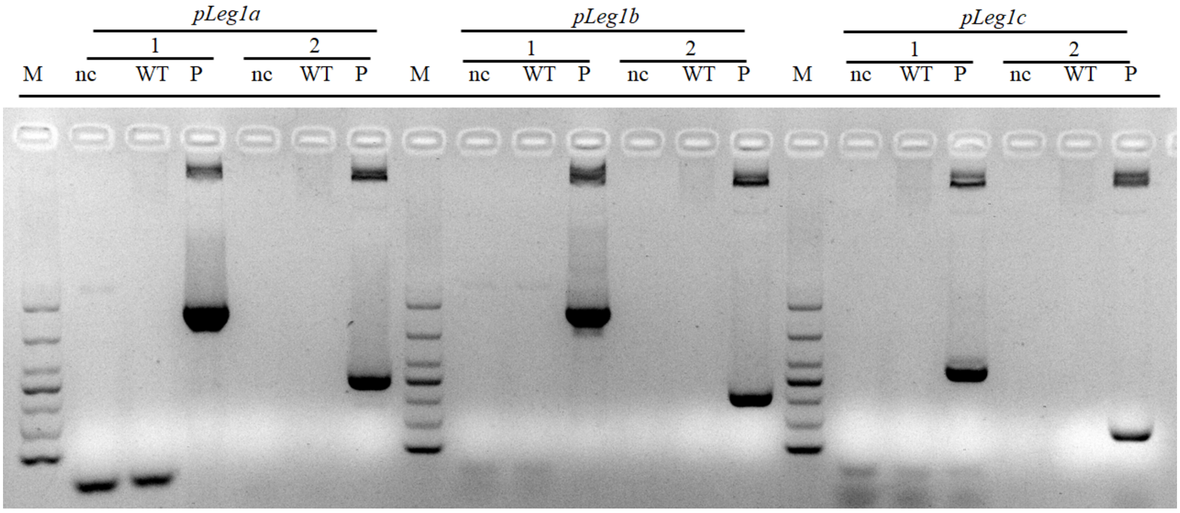

**Fig. S4** Clustering analysis of LEG1 proteins from representative vertebrates using structural information.

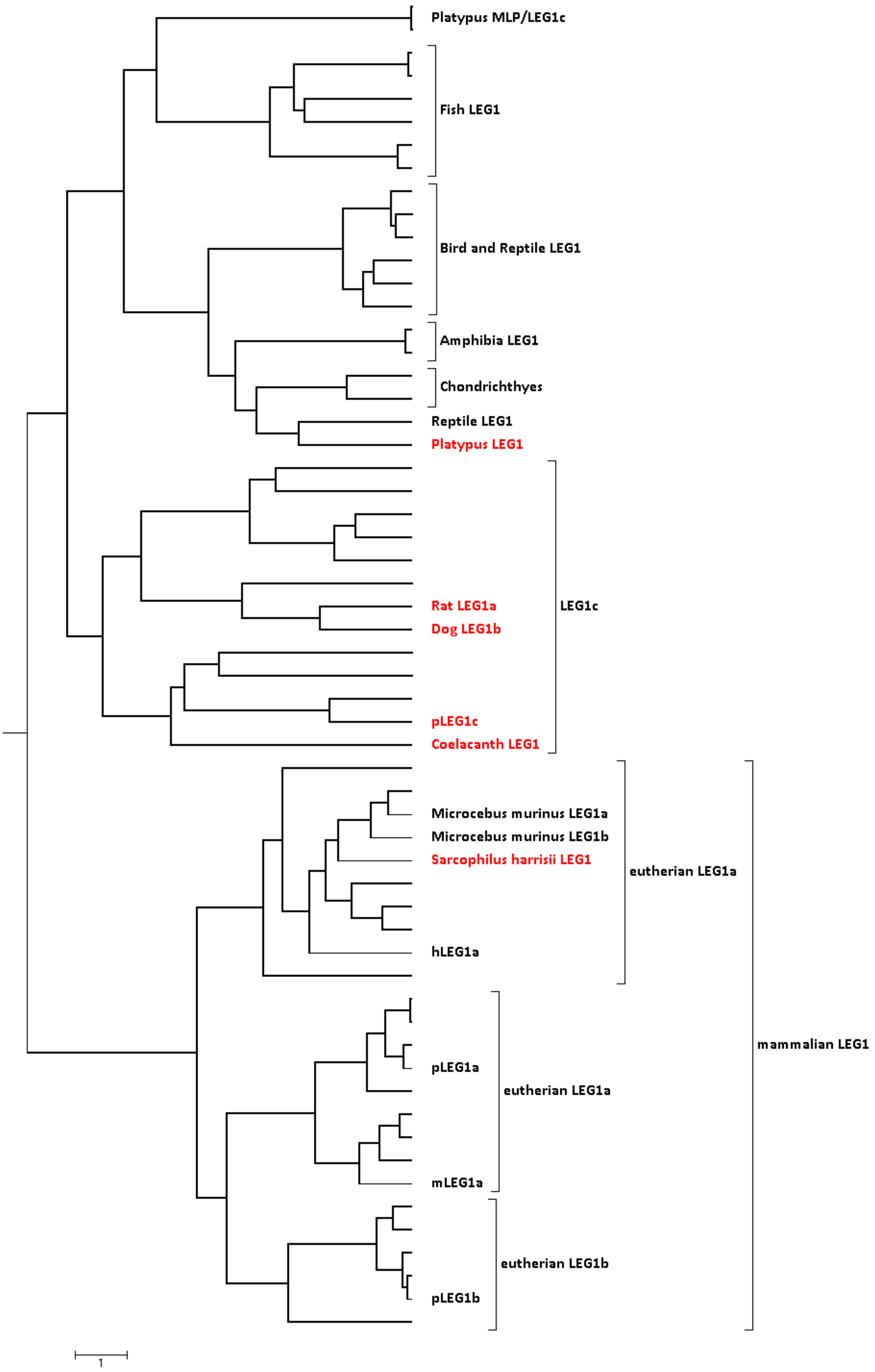

Platypus MLP/LEG1c
Fish LEG1
Bird and Reptile LEG1
Amphibia LEG1
Chondrichthyes
Reptile LEG1
Platypus LEG1
Rat LEG1a
LEG1c
Dog LEG1b
pLEG1c
Coelacanth LEG1
Microcebus murinus LEG1a
Microcebus murinus LEG1b
Sarcophilus harrisii LEG1
eutherian LEG1a
hLEG1a
pLEG1a
eutherian LEG1a
mammalian LEG1
mLEG1a
eutherian LEG1b
pLEG1b
1

**Supplementary Table S1**. dN/dS calculation result for *Leg1* paralogs.

| Name | paralog comparison | dN/dS value | | |
|---|---|---|---|---|
| | | GY-HKY | YN | GYN |
| Microcebus murinus | leg1a vs leg1b | 0.449084 | 0.419354 | 0.389294 |
| Myotis lucifugus | leg1a vs leg1b | 0.452543 | 0.410929 | 0.379824 |
| | leg1a vs leg1c1 | 0.380434 | 0.302643 | 0.400732 |
| | leg1a vs leg1c2 | 0.345992 | 0.357409 | 0.441831 |
| | leg1b vs leg1c1 | 0.447599 | 0.471695 | 0.596222 |
| | leg1b vs leg1c2 | 0.477402 | 0.46918 | 0.426558 |
| | leg1c1 vs leg1c2 | 1.63797[$] | 1.72524 | 1.72804 |
| Bos taurus | leg1a vs leg1c | 0.358856 | 0.318116 | 0.418668 |
| Felis cattus | leg1a vs leg1b | 0.414099 | 0.35442 | 0.315877 |
| Canis lupus familiaris | leg1a vs leg1b | 0.375504 | 0.380175 | 0.347006 |
| Loxodonta africana | leg1a1 vs leg1a2 | 0.982733 | 0.996845 | 0.994132 |
| | leg1a1 vs leg1b | 0.412994 | 0.362258 | 0.380684 |
| | leg1a2 vs leg1b | 0.540912 | 0.451968 | 0.411972 |
| Equus caballus | leg1a vs leg1b | 0.58754 | 0.620634 | 0.595239 |
| Sus scrofa | leg1a vs leg1b | 0.563512 | 0.605614 | 0.586966 |
| | leg1a vs leg1c | 0.380957 | 0.45261 | 0.612616 |
| | leg1b vs leg1c | 0.380957 | 0.45261 | 0.612616 |
| Oryctolagus cuniculus | leg1a1 vs leg1a2 | 0.416805 | 0.413494 | 0.396333 |
| | leg1a1 vs 1b | 0.403323 | 0.38284 | 0.34616 |
| | leg1a1 vs 1c1 | 0.347461 | 0.3213 | 0.413047 |
| | leg1a1 vs 1c2 | 0.479589 | 0.751756 | 0.821352 |
| | leg1a2 vs leg1b | 0.40218 | 0.358149 | 0.318449 |
| | leg1a2 vs leg1c1 | 0.323505 | 0.173862 | 0.197908 |
| | leg1a2 vs leg1c2 | 0.571378 | 0.88596 | 0.895714 |
| | leg1b vs leg1c1 | 0.323505 | 0.173862 | 0.197908 |
| | leg1b vs leg1c2 | 0.525019 | 0.334201 | 0.500269 |
| | leg1c1 vs leg1c2 | 3.0238 | 2.87337 | 2.87337 |
| Rattus norvegicus | leg1a vs leg1c1 | 0.505463 | 0.287054 | 0.348889 |
| | leg1a vs leg1c2 | 0.748992 | 0.676924 | 1.08021 |
| | leg1c1 vs leg1c2 | 1.16422 | 1.22064 | 1.2297 |
| Ovis aries | leg1a leg1c | 0.380942 | 0.313009 | 0.410284 |
| platypus | leg1 vs leg1c | 0.376625 | 0.179801 | 0.205146 |
| Sarcophilus harrisii | leg1 vs leg1c | 0.451711 | 0.503015 | 0.666571 |
| Oncorhynchus mykiss | leg1.1 vs leg1.2 | 0.289064 | 0.283553 | 0.274562 |
| Danio rerio | leg1.1 vs leg1.2 | 1.03808 | 1.09409 | 1.0929 |

[$] The red highlighted numbers indicate non-significant P-values of Fisher's exact test.

**Supplementary Table S2.** Oligos used in the study.

| | Primer | Sequence |
|---|---|---|
| Coding sequence amplification | pLeg1a-1-F | ATGGCTTTCCTTCCTCCTTGG |
| | pLeg1a-1-R | GTGAACCTGTAGCGTCATCCA |
| | pLeg1b-1-F | ATCTTCCCTGGGCCTGTGTA |
| | pLeg1b-1-R | TCCTTGCAACTTTGAACTTCATT |
| | pleg1c-F | ATGTACCAAGTTTCAAATGG |
| | pleg1c-R | AATTGCGTTTAATCATGGAA |
| 5’ & 3' RACE | 3RACE OligodT | CAAGCTGGTCCTGGACATCGAGATCACC ACCTACCGCAAGCTTTTTTTTTTTTTTT |
| | 3RACE L1 | CAAGCTGGTCCTGGACATC |
| | 3RACE L2 | GATCACCACCTACCGCAAG |
| | pleg1a-3RACEGSP1 | AATCACGCTTTTGCCACCAC |
| | pleg1a-3RACEGSP2 | GTTCCAGAAGGCCCTACCAC |
| | pleg1a-5RACEGSP1 | TGGGTGGTGGCAAAAGCGTGA |
| | pleg1a-5RACEGSP2 | ACCAGCTGTCCACAGAGATA |
| RT-PCR | pLeg1a-1-F | ATGGCTTTCCTTCCTCCTTGG |
| | pLeg1a-1-R | GTGAACCTGTAGCGTCATCCA |
| | pLeg1a-2-F | CTTTTGCCACCACCCAAGGA |
| | pLeg1a-2-R | CGGTGTTCTGAAGGTCGGTAA |
| | pLeg1b-1-F | ATCTTCCCTGGGCCTGTGTA |
| | pLeg1b-1-R | TCCTTGCAACTTTGAACTTCATT |
| | pLeg1b-2-F | TCCTTGCTGCGGTTGATTCT |
| | pLeg1b-2-R | CACAAACAAGGCCCAGCTTC |
| | pleg1c-1F | GGCATCGGCTTCTATGTGCT |
| | pleg1c-1R | AGTGGCCTATGTGGGAAACC |
| | pleg1c-2F | ATGTCCATCAAGCAGCCGTT |
| | pleg1c-2R | ACGATCTTGGTCTGTGAGTGG |
| | GAPDH-F | GGGCATGAACCATGAGAAGT |
| | GAPDH-R | AAGCAGGGATGATGTTCTGG |
| qRT-PCR | pLeg1a-qPCR-F | GGATGCACACATTTCAGCCT |
| | pLeg1a-qPCR-R | ACAAGGACTCGTGGTGGTAG |
| | pLeg1b-qPCR-F1 | CCCTCTTCCCTACGACTTTGA |
| | pLeg1b-qPCR-R1 | AACAAGCATTCGTGTTGGC |
| | pleg1c-qPCR-F | GCATCGGCTTCTATGTGCTT |
| | pleg1c-qPCR-R | TGCCTCTATCCATAGCTCGTG |
| | -qPCR-f | ATCACCATCTTCCAGGAGCGA |
| | gapdh-qPCR-r | AGCCTTCTCCATGGTCGTGAA |
| Plasmid construction | BamHI-Kozakpleg1a-F | CTCAGTGGATCCGCCGCCAT GGCTTTCCTTCCTCCTTGG |
| | pleg1a-express-R | TTGTCTCGAGGAAATGTTGGAATGCTGCAA |
