# Supplementary material for: Evolutionary and Molecular Characterization of liver-enriched gene 1
Source: Sci Rep. 2020 Mar 6;10:4262. doi: 10.1038/s41598-020-61208-7 (PMC7060313; doi:10.1038/s41598-020-61208-7)
Supplement: Supplementary file 2 [file 41598_2020_61208_MOESM2_ESM.docx]

**Title: Evolutionary and Molecular Characterization of *liver-enriched gene 1***

**Authors:** Yanna Dang^1^, Jin-Yang Wang^1^, Chen Liu^1^, Kun Zhang^1^, Peng Jinrong^1^, Jin He^1*^

**Affiliations**

^1^ Department of Animal Science, College of Animal Sciences, Zhejiang University, Hangzhou, PR China

^*^Corresponding author: hejin@zju.edu.cn


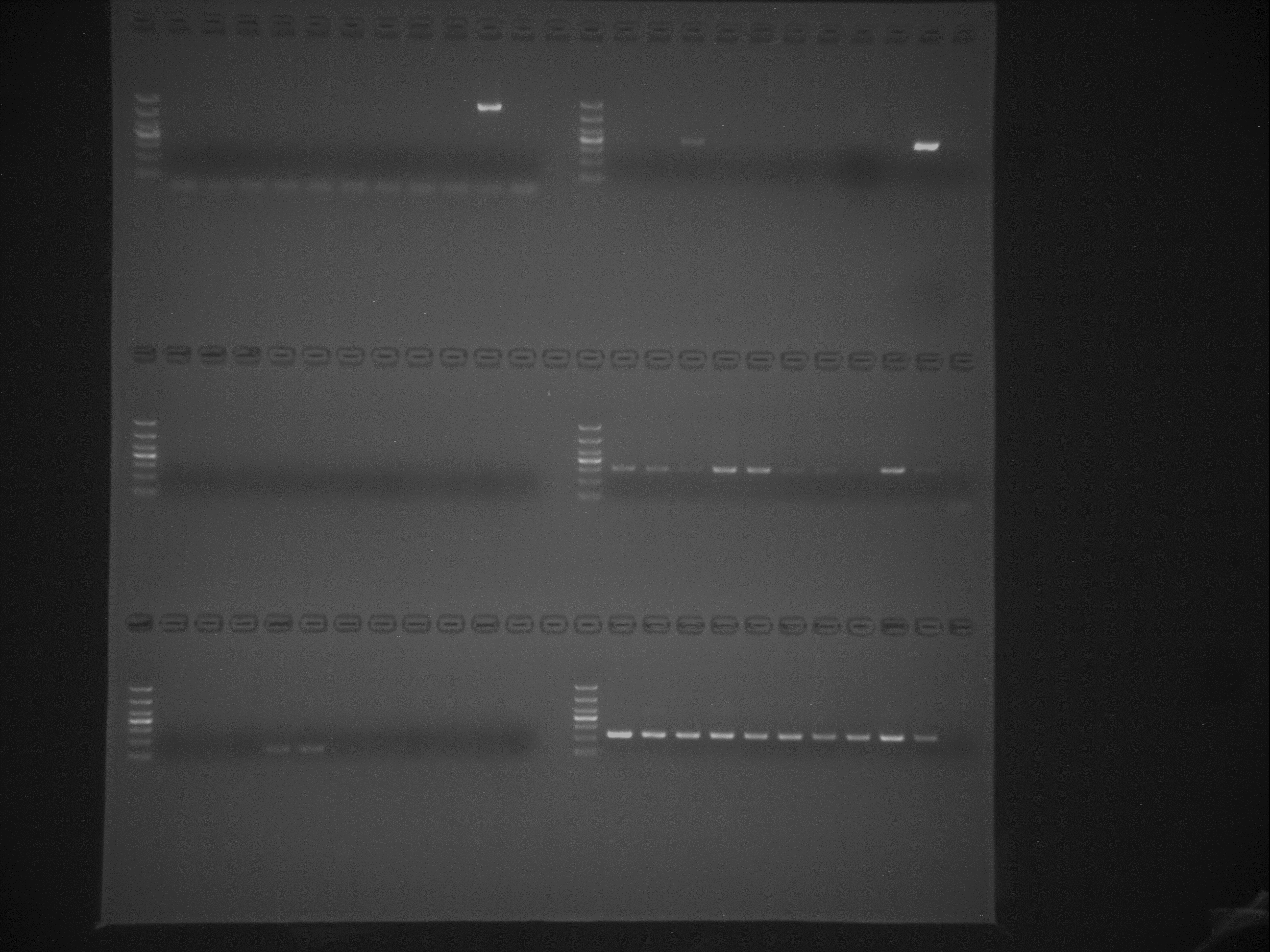


This image is the original file for Figure 4A. Up left 12 lanes are the RT-PCR result for pLeg1a. Middle left 12 lanes are the RT-PCR result for pLeg1b. Bottom right 12 lanes are the RT-PCR result for pLeg1c. The others are irrelevant results.


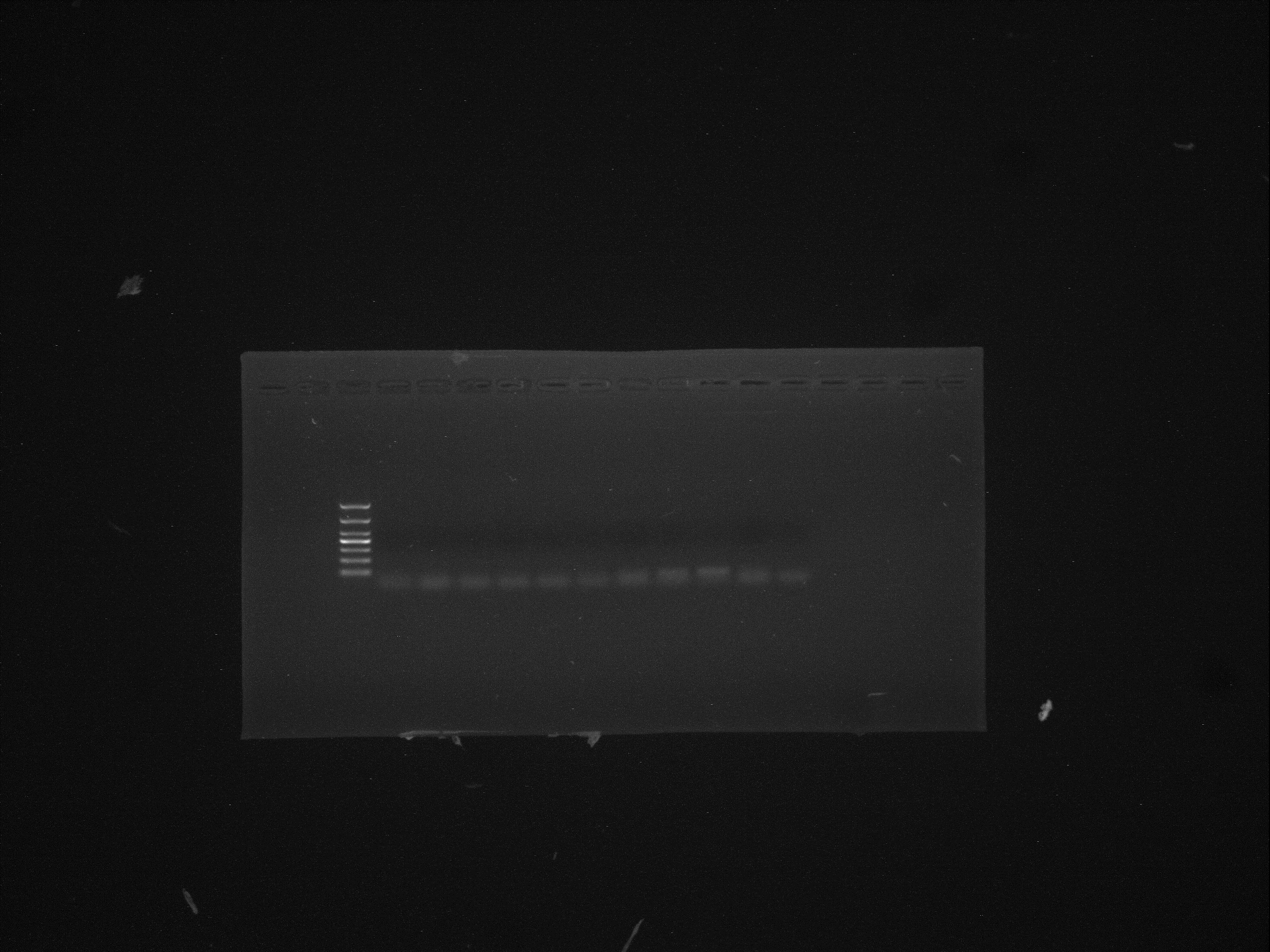


This image is the original result of pLeg1c in Figure 4A.


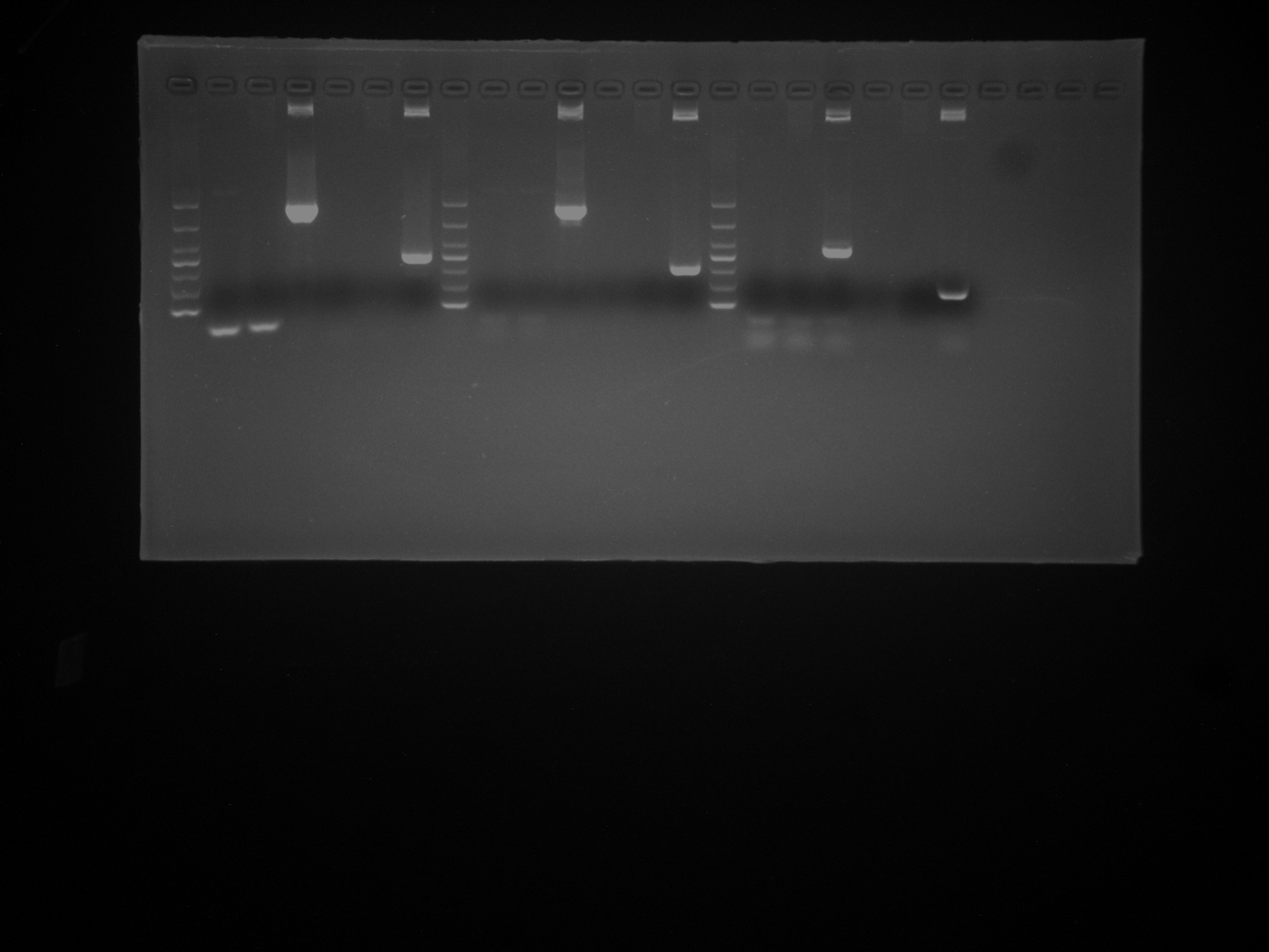


This is original uninverted image for Figure S3.
